# Expression of stress responsive genes enables *Limosilactobacillus reuteri* to cross-protection against acid, bile salt, and freeze-drying

**DOI:** 10.3389/fmicb.2024.1437803

**Published:** 2024-09-30

**Authors:** Zhenzhen Liu, Xiao Zhao, Hina Iqbal Bangash

**Affiliations:** ^1^Antibiotics Research and Re-evaluation Key Laboratory of Sichuan Province, Sichuan Industrial Institute of Antibiotics, School of Pharmacy, Chengdu University, Chengdu, China; ^2^State Key Laboratory of Agricultural Microbiology, Hubei Hongshan Laboratory, College of Life Science and Technology, Huazhong Agricultural University, Wuhan, China

**Keywords:** *Limosilactobacillus reuteri*, acid stress, bile salts stress, freeze-drying, cross-protection

## Abstract

**Introduction:**

*Limosilactobacillus reuteri* effectively colonizing the gut, secretes antimicrobial compounds and strengthens immune system function. Considering these health benefits, increasing its stress assessments efficiency could improve its commercial viability.

**Methods:**

In this work, the resistance of *L. reuteri* FP41 to acid, bile salts, and freeze-drying was examined.

**Results:**

The findings showed that strain FP41 demonstrated a strong resistance to acid/bile salt stresses. The transcriptome revealed a significant up-regulation of various stress response genes, including those related to membrane integrity, glutamine metabolism, OsmC family protein, ABC transporters, and chaperonin. Subsequent research demonstrated that overexpression of three stress response-specific proteins, including glutamate decarboxylase GatD, osmotically induced bacterial protein OsmC, and membrane protein component CsbD, significantly increased the survival rate of *L. reuteri* Z204 under acid/bile salts stress. Notably, overexpression of the OsmC, CsbD, and GatD proteins also enhanced the survival of *L. reuteri* after freeze-drying.

**Discussion:**

The development of a unique cross-protection method is highlighted in this study, that might significantly increase cellular resistance to acid, bile salts, and cold stresses. This finding could significantly impact the way that *L. reuteri* is employed in industrial manufacturing processes.

## Introduction

Probiotics are known as “live microorganisms that, when administered in adequate amounts, confer a health benefit on the host.” Through consumption of fermented foods or powdered probiotic supplements, lactic acid bacteria (LAB), a type of bacteria that can produce significant levels of lactic acid, are commonly employed as probiotics to improve immunity and promote health (Daba and Elkhateeb, 2020). Bacteria are exposed to a variety of extreme environmental conditions throughout the production and utilization of LAB, including osmotic pressure, temperature, oxidation stress, and starvation (Mbye et al., 2020). Bacteria encounter challenges in the gastrointestinal tract due to low pH and high osmotic pressure caused by stomach acid and bile salts. Probiotics, on the other hand, need to overcome these stresses in order to colonize and function effectively in the human intestine. The phospholipids and proteins in the cell membranes of bacteria in the gastrointestinal system can be disrupted by exposure to bile salts, which can impair their ability to survive and disturb their cellular homeostasis (Ruiz et al., 2013). The favored approach for long-term preservation in strain resource centers and production industries is now freeze-drying due to its more effective long-term cell survival rate and ease of preservation, transportation, and application (Liu Y. et al., 2022). Bacteria are exposed to multi-stresses throughout the freeze-drying process, including cold, desiccation, crystallization, osmotic stress and others. These stresses have the ability to alter the structure of cell membrane and cellular physiology, which may lower bacterial activity (Gao et al., 2022). But its understanding of how exactly LAB responds to these stressors is still limited.

Extreme environmental stimuli usually have an impact on a wide range of biological functions in bacteria. Adaptive mechanisms, including alterations to protein expression, can be induced by sublethal environmental stresses, enabling them to withstand harsh environments (Mbye et al., 2020). For example, the expression of stress response proteins HSPs, under harsh settings may enhance the probiotics’ ability to withstand heat during food processing and boost their survival rate after freeze-drying (Liu et al., 2015; Chen et al., 2017). It is now commonly recognized that lactic acid rapidly permeates the cytoplasmic membrane, dissociates into protons to cause intracellular acidification, and ultimately causes damage to DNA and proteins, all of which negatively affect LAB (Shabala et al., 2006). The ATP synthase subunit beta and chaperone protein DnaK was used to against the acid stress in *L. pentosus* (Pérez Montoro et al., 2018). Modulation membrane fluidity, protecting and repairing macromolecules, strengthening the proton pump, controlling enzyme and metabolic activity, and consuming an excessive amount of cytoplasmic protons can all help reduce these negative effects (Gao et al., 2022). LAB possesses several established bile salts tolerance mechanisms, including stress protein synthesis, alterations to cell membranes, and bile salts metabolism (Bagon et al., 2021; Ayyash et al., 2021). It’s worth noting that resistance to certain stresses as well as unrelated stresses that improve overall tolerance for multiple environmental stresses can cross-protect probiotic survival in LAB (Gao et al., 2022). Accordingly, in order to minimize stress-related damage and improve survival rates, it is crucial to gain a thorough understanding of how LAB responds to environmental stresses. It is essential for the screening and selection of candidate probiotic strains (Mbye et al., 2020).

Almost all vertebrates and other animals have the heterotypic fermentative bacterium *Limosilactobacillus reuteri* in the gastrointestinal tracts, which serves a variety of health-beneficial functions for its host (Mu et al., 2018). Crucial steps towards expanding *L. reuteri’*s industrial application include investigating its stress response system and enhancing its adaptability through genetic modification. In this study, we utilized transcriptome analysis to identify the critical genes that *L. reuteri* FP41, a highly viable and resistant bacterium isolated from the intestine of a healthy pig, responds to acid/bile salt stress. By assessing the survival rate under the acid/salts stress after these observed stress-related genes were overexpressed, the mechanism underlying *L. reuteri’*s resistance to these stresses was studied. Furthermore, the identified genes were investigated for their potential role in *L. reuteri’s* cross-protection mechanism and freeze-drying tolerance. This work provides new insights into the processes by which *L. reuteri* responds to environmental stress and establish a framework for future research on the utilization of cross-protection mechanisms in stress response.

## Materials and methods

### Strains, cultural conditions, and transformation

*L. reuteri* strains FP41 (MGSC70001), FP42, and Z204 were isolated from the feces of pigs (The fecal samples were gradient-diluted with sterile water, covered with MRS Plates, and single colonies were chosen and repeatedly emphasized before being screened and identified.), *L. reuteri* strains ATCC 23272 was obtained from ATCC. The DH5α strain of *Escherichia coli* is used for gene cloning. *E. coli* strains was cultured in LB medium with or without 100 μg/mL Ampicillin in order to screen positive clones (Coolaber, Beijing, CN). The strains of *L. reuteri* ATCC23272, FP41, FP42, and Z204 were cultured at 37°C in MRS medium.

The construction of overexpression plasmids was based on pMG36e. These genes were amplified from *L. reuteri* strains FP41 genomic DNA using Phanta DNA Polymerase (Vazyme, Nanjing, China), and the primers listed in Supplementary Table S1. The restriction enzyme XbaI/NotI was used to digest the *osmC*, *csbD*, and *cobQ* gene segments that were produced using PCR and plasmid pMG36e over night at 37°C. The digested products were subsequently transformed into DH5a after being exposed to T4 ligase for 2 hours at 22°C. After an overnight culture, transformants were chosen, PCR analysis and sequence were used to confirm positive clones. Plasmids from positive clones were extracted and being electroporated into *L. reuteri* 204. For *L. reuteri* strains’ electroporation (Liu et al., 2023; Guo et al., 2023), 500 ng plasmids were added to 80 μL competent cells and electroporated at 2,500 V, 25 μF, and 200 *Ω*. The cuvette was immediately put in an ice bath for 5 min after electroporation and filled with prechilled modified MRS liquid medium supplemented with 171.15 g/L sucrose (Sinopharm, Beijing, CN). Following a 6 h incubation at 37°C, the cells were centrifuged and the remaining bacterial solution was resuspended in 100 μL. The resuspended solution was spread on an agar plate containing MRS medium with 5 μg/mL erythromycin (Coolaber, Beijing, CN), and incubated at 37°C for 2 days.

### Growth curves test

Every two hours, the OD_600_ value was measured while the 1% overnight-cultured *L. reuteri* was added to MRS liquid medium and incubated at 37°C. The culture was diluted and plated onto solid MRS media after a 12 h fermentation. After that, the plates were incubated for 48 h at 37°C to count the viable bacteria. The pH of MRS was adjusted to 2.0, 3.0, 4.0, 5.0, 6.0 and 6.8 to obtain the acid tolerance growth curves; on the other hand, the addition of pig bile salt (0.02, 0.04, 0.06, and 0.08%) to MRS generated the bile salt tolerance growth curves.

### RNA library construction, quantification, and transcriptome analysis

The quality and the quantity of the extracted total RNA were determined by Nano Photometer spectrophotometer and Qubit 2.0 Fluorometer. RNA was examined for integrity by Agilent 2,100 bioanalyzer before being used as input material for cDNA library preparations.

HiScript III RT SuperMix for qPCR (Vazyme, Nanjing, CN) was used to reverse transcribed 1 μg of each total RNA sample in order to synthesis cDNA for RT-qPCR (Conesa et al., 2016). Utilizing the Taq Pro Universal SYBR qPCR Master Mix (Vazyme, Nanjing, CN), real-time qPCR was performed out in an ABI Real-Time PCR Instrument. Supplementary Table S1 lists the amplified targets’ primer sequences.

Using the RIBO-Zero kit, rRNA was extracted to enrich the mRNA, which was then fragmented and used as a template for cDNA synthesis with random hexamers for transcriptome analysis. The double-stranded cDNA was purified using AMPure XP Beads and the USER enzyme was used to remove the second strand containing Uracil. Using an Illumina HiSeq2000 instrument, the cDNA end was repaired, polyA tail was added, splice sequenced and length screened. Before aligning the raw data to the reference genome sequence of *L. reuteri* I5007, adapter sequences and low-quality bases were removed in order to obtain clean reads (Powell et al., 2012). For rapid genome-to-sequencing data comparison, the Burrows-Wheeler Transform (BWT) algorithm with Ferragina-Manzini (FM) indexes was used. The reads mapped to each gene were then counted using the HTSeq software, and per kilobase per million mapped reads (RPKM) was then calculated based on gene length and the number of mapped reads (Chang et al., 2009; McClure et al., 2013). We expressed transcript expression levels using RPKM in order to determine the levels of gene expression in different groups. Significant differences in gene expression were analyzed using the DESeq2 software (Kanehisa et al., 2004). To identify differentially expressed genes (DEGs), a *p*-value>0.05 and a log foldchange (logFC) < 2 were established as criteria. Transcriptome data were deposited in the SRA database under Accession number PRJNA1032396.

### Acid tolerance test

The artificial gastric juice (Pedersen et al., 2004) contained: 3.50 g/L glucose, 2.05 g/L NaCl, 0.11 g/L CaCl_2_, 0.37 g/L KCl, 0.60 g/L KH_2_PO_4_, 0.05 g/L pig bile salt (Sinopharm, Beijing, CN); 8.30 g/L peptone (OXOID, United Kingdom), 0.10 g/L Lysozyme (Amresco, United States), 13.30 mg/L Pesin (Macklin, Shanghai, CN), and the pH was adjusted to 2.5 for sterilization and backup use. 1 mL of *L. reuteri* overnight cultures was added into 9 mL artificial gastric juice, and cultured in a 37°C incubator. Viable bacterial count was measured by sampling at 0, 1, and 3 h, respectively. The equation for survival rate is as follows: survival rate = number of bacteria after treatment / number of bacteria in 0 h before treatment * 100%.

### Bile tolerance test

MRS media containing 0.1, 0.3 and 0.5% (w/v) of pig bile salt were prepared, respectively (Nithya and Halami, 2012). One ml of *L. reuteri* overnight cultures was inoculated into 9 mL of pig bile solution at different concentrations, and the mixture was then incubated at 37°C for 12 h. Samples were taken for dilution plate counting at the 0, 12 h. Viable bacteria count was measured. The equation for survival rate is as follows: survival rate = number of bacteria after treatment / number of bacteria in 0 h before treatment * 100%.

### Freeze drying test

The bacteria were centrifuged for 10 min at 8, 000 g, 4°C. The bacterial pellet was collected and the supernatant was removed. The bacteria suspension was mixed with the protection agent solution (10% skimmed milk powder, 6% maltose, 1.5% mannitol and 0.8% glycine) at a volume ratio of 1:9 after the bacteria mud and water were mixed at a mass ratio of 1:1. 1 mL was taken and divided into the freeze-drying bottle once it was completely homogenous. The procedures for freeze drying were as follows: pre-freeze for 5 h at −80°C, followed by freeze drying for 12 h at −50°C and 0.25 mba of vacuum pressure. The equation for survival rate is as follows: survival rate = number of bacteria after treatment / number of bacteria in 0 h before treatment * 100%.

### Proteome analysis

Proteins were extracted from the overnight cultured sample by lysing it in SDT buffer (4% SDS, 100 mM Tris–HCl, pH 7.6). After filter-aided sample preparation (FASP), protein digestion was performed. For peptide quantification (OD280), the resulting peptide digests from each sample were desalted using C18 Cartridges, lyophilized, and reconstituted in 40 μL of 0.1% (v/v) formic acid. Using a timsTOF Pro mass spectrometry system (Bruker) coupled to Nanoelute (Bruker), LC–MS/MS analysis was conducted. After loading the peptides onto a C18-reversed phase analytical column (Thermo Scientific Easy Column, 25 cm long, 75 μm inner diameter, resin particle size: 1.9 μm) with an initial composition of 95% buffer A (0.1% Formic acid in water), followed by separated using a linear gradient of buffer B (99.9% acetonitrile and 0.1% Formic acid) at a flow rate of 300 nL/min. With a 1.5 kV electrospray voltage applied, the mass spectrometer operated in positive ion mode. The TOF detector analyzed precursor ions and fragments within the mass range of m/z 100–1700. The timsTOF Pro was utilized in the parallel accumulation serial fragmentation (PASEF) (Meier et al., 2018) mode, and the subsequent parameters were followed for data collection: 1 MS and 10 MS/MS PASEF scans were performed with an ion mobility coefficient (1/K0) value ranging from 0.6 to 1.6 *Vs* cm^2^, and active exclusion was enabled with a 24 s release time. MaxQuant (Cox and Mann, 2008) version 1.6.14 software was used for combining and analyzing the MS raw data for each sample (Yu et al., 2004; Finn et al., 2016; Schwanhäusser et al., 2011; Cox et al., 2011). The MS data have been deposited to the ProteomeXchange Consortium via the iProX repository with the data set identifier IPX0007438000. The DEGs are listed in Supplementary Table S2.

### Statistical analysis

All statistical analyses were performed using GraphPad Prism 8 and SPSS. One-way analysis of variance (ANOVA) was used to evaluate the data, and then Tukey’s back testing was performed to determine the statistical difference between each group.

## Results and discussion

### Analysis of *Limosilactobacillus reuteri* growth characteristics and the acid/bile tolerance

As a probiotic, *L. reuteri*’s tolerance to low pH and high bile salts is vital for its survival in gastric juice and the intestines. The starting strains for further stress resistance studies were selected based on the growth characteristics and acid/bile tolerance of *L. reuteri*.

**Figure 1:**
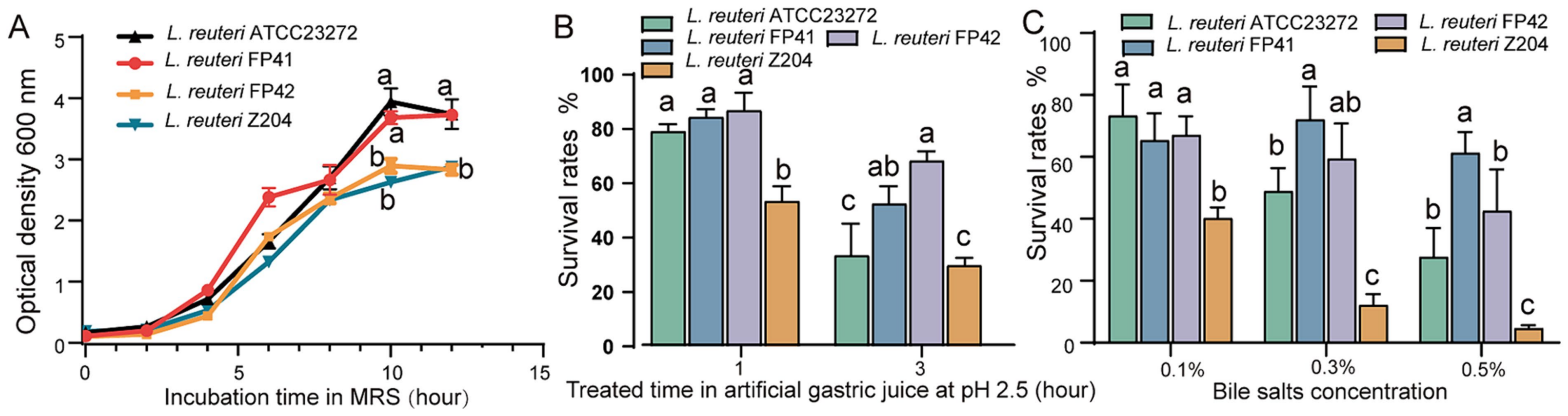
The growth characteristics and the acid/bile tolerance in *L. reuteri*. **(A)** Growth curves of FP41, FP42, Z204 and ATCC23272 in MRS based on absorbance at 600 nm. Survival rates of FP41, FP42, Z204 and ATCC23272 in pH 2.5 artificial gastric juice treatment **(B)** and different pig bile salts concentration treatment **(C)**. Error bars: standard derivations of three independent experiments. Data with different superscript letters (a, b, and c) are significantly different (*p* < 0.05) according to one-way ANOVA followed by Tukey’s test.

According to the findings, the strains of *L. reuteri* FP41, FP42, Z204 and ATCC 23272 showed the highest growth with OD600 values of 3.73, 2.83, 2.88 and 3.94, respectively (Figure 1A), and with the highest viable counts were 9.15 × 10^8^ CFU/mL, 4.55 × 10^8^ CFU/mL, 7.10 × 10^8^ CFU/mL and 12.98 × 10^8^ CFU/mL, respectively. These results indicate that, while growing similarly to type strain ATCC 23272, the candidate strain FP41’s growth curve was significantly better than FP42 and Z204. Strong acid tolerance was demonstrated by *L. reuteri* FP41, FP42, ATCC 23272 and Z204, as they were able to survive in gastric juice with a pH of 2.5 (Figure 1B), suggesting their advantages in passing through the human digestive tract (pH 1.5–7.5) (Fallingborg, 1999). Furthermore, FP41 and FP42 showed a significantly higher survival rate than ATCC 23272 and Z204 at 3 h upon acid treatment. Even at a concentrations of 0.5%, *L. reuteri* FP41, FP42 and ATCC23272 all shown good tolerance to bile salts (Figure 1C), suggesting their advantages in passing through the human small intestine (Begley et al., 2005). When treated with pig bile salts at concentration of 0.3 and 0.5%, FP41 had a significantly higher survival rate than ATCC23272, which is similar to acid tolerance. Furthermore, compared to other strains, Z204 showed a markedly lower bile salt tolerance, even at a bile salts concentration of just 0.1%. These findings led us to choose FP41 as the transcriptome analysis strain and Z204 as the host for the overexpression of stress proteins.

### Transcriptome sequencing of *Limosilactobacillus reuteri* acid/bile salts stress

**Figure 2:**
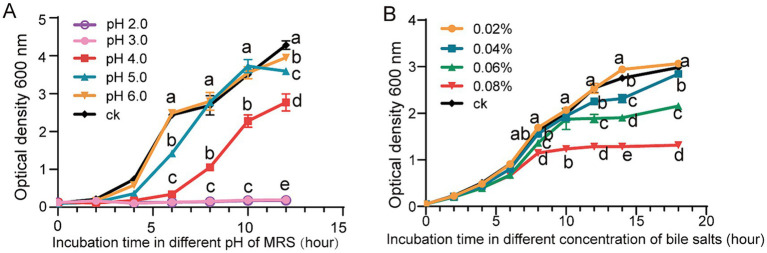
The growth characteristics and the acid/bile tolerance in *L. reuteri.* The growth curves of *L. reuteri* FP41 in MRS under different pH values **(A)** and different bile salts concentrations **(B)**. Error bars: standard derivations of three independent experiments. Data with different superscript letters (a, b, c, d, and e) are significantly different (*p* < 0.05) according to one-way ANOVA followed by Tukey’s test.

The growth of *L. reuteri* FP41 was examined under various pH conditions or variable concentrations of bile salts in order to determine an appropriate sample period and treatment conditions. The results showed that growth was significantly slower at pH 4.0 than it was for the control group at pH 6.8, that adjusting the pH to 2.0 and 3.0 successfully inhibited the growth of bacteria (Figure 2A). Bile salts inhibited *L. reuteri* FP41 growth gradually; the greatest effect was observed at a concentration of 0.08% (Figure 2B). For RNA-sequencing, *L. reuteri* FP41 was thus cultivated in MRS at pH 4.0 or 0.08% bile salts concentration till OD600 = 1.0.

### The stress response genes expression in *Limosilactobacillus reuteri* under acid/bile salts

A total of 233 genes were significantly differentially expressed in the pH 4.0 group, of which 126 genes were up-regulated compared with the control group, and 607 genes were significantly differentially expressed in the bile salts stress group, of which 315 genes were up-regulated compared with the control group, according to transcriptome data analysis (Supplementary Table S1). Among the genes that have been significantly up-regulated are cytochrome, glutamine metabolism, OsmC family protein, ABC transporter system, and integral component of membranes (Table 1; Supplementary Table S1). Under stress of acid and bile salts, most genes were up-regulated concurrently, and the highest number of genes related to the membrane was seen. This finding aligns with earlier research indicating that probiotics largely alter cell membranes to enhance their ability to withstand acidic or bile salt environments (Ayyash et al., 2021; van Der Heide and Poolman, 2000). The cytoplasmic membrane is a major barrier to most solutes and is sensitive to stress (Spano and Massa, 2006), which is consistent with our findings indicating the upregulation of a significant number of genes related to membranes following treatment with acid/bile salt (Table 1; Supplementary Table S1). Particularly, responses to various stresses, such as heat, acid, oxidative stress, phosphate starvation, and salt stress, are mediated by the membrane protein CsbD (Han et al., 2017; Prágai and Harwood, 2002). In our study, we found that under acid/bile salts stress, CsbD was markedly upregulated (Table 1). Furthermore, in response to acid stress, bacteria can produce or consume ammonia by upregulating certain genes associated with glutamine metabolism (Lu et al., 2013). This is consistent with our findings, as glutamine metabolism-related gene *gatD* was similarly up-regulated (Table 1). These findings imply that *L. reuteri* might have an ammonia cycle to deal with acid stress, and that the ammonia molecule that results is directed at the MurT active site. Notably, under acid/bile salts stress, OsmC, a thiol-dependent organic hydroperoxide reductase (Ohr), showed significant up-regulation (Table 1). In *Corynebacterium glutamicum* (Si et al., 2019), as well as in peroxide metabolism and preservation against oxidative stress in *mycobacteria* (Saikolappan et al., 2011), the protein OsmC plays a critical protective role against organic hydroperoxides (OHPs) stress. Similar results were found in the transcriptome data (Supplementary Table S1), which is consistent with previous findings that ABC transporters are positively associated with acid tolerance in *L. lactis* (Zhu et al., 2019) and that chaperonin pumps significantly contribute to resistance against acid and bile salts stress (Gao et al., 2022; Zhang et al., 2017).

**Table 1:** Transcriptional changes (log2-fold) of genes response to acid/bile salts stress in *L. reuteri* FP41 according to the transcriptome data.

Gene ID	pH 4.0 *vs* control	0.08% Bile salts vs. control	Annotation
Integral component of membrane			
LRI_RS02215	1.86	3.17	CsbD family protein
LRI_RS02220	1.71	2.09	GlsB/YeaQ/YmgE family stress response membrane protein
Glutamine metabolism			
LRI_RS10405	1.05	2.59	Glutamine amidotransferase GatD (CobQ)
OsmC family protein			
LRI_RS06390	1.93	3.51	OsmC family protein

### The construction of the overexpression plasmid

**Figure 3:**
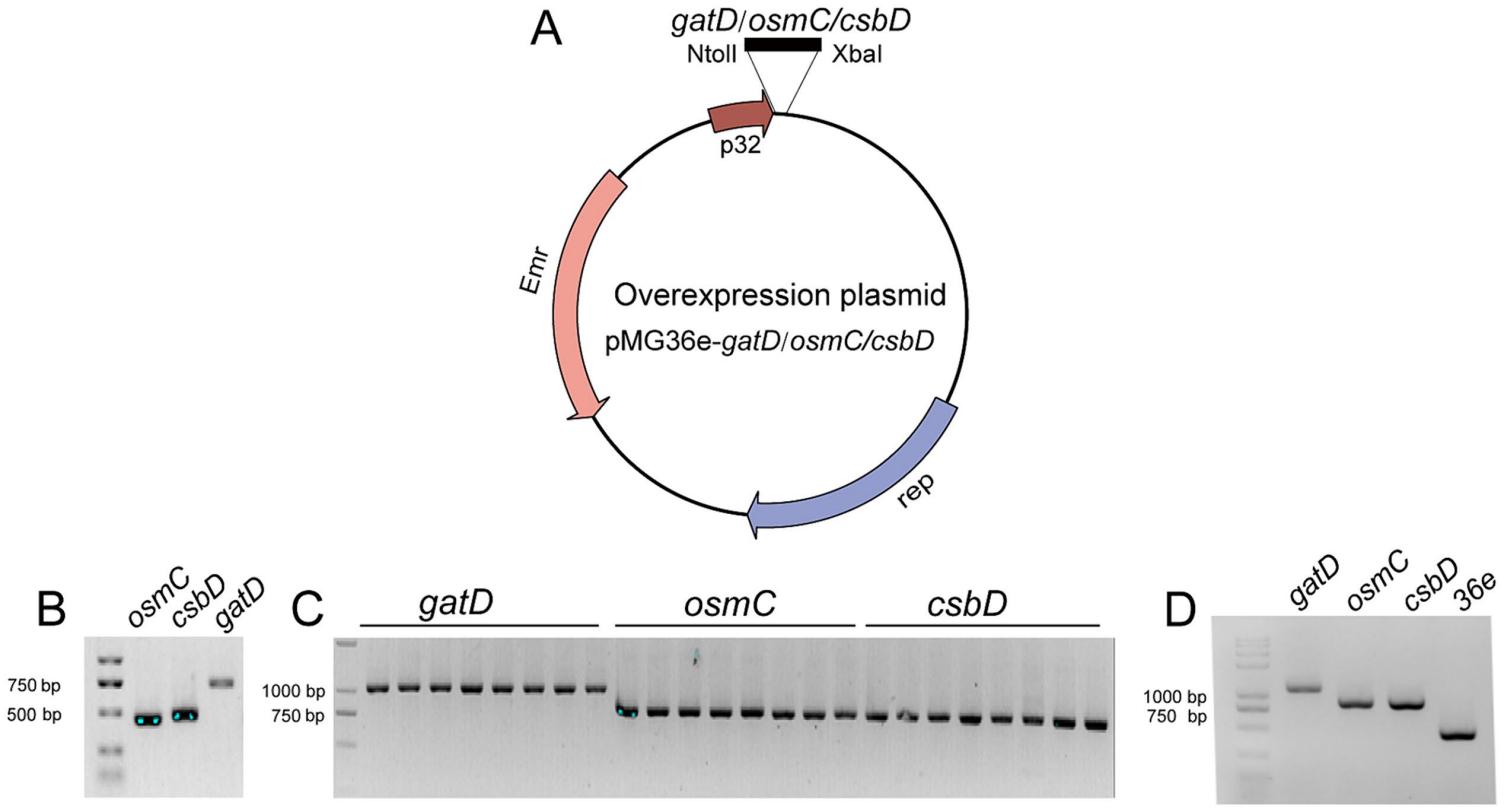
Schematic representation of the construction of the overexpression plasmid. **(A)** The plasmid map of pMG36e-gatD/osmC/csbD. **(B)** PCR amplification of these genes from *L. reuteri* strains FP41 genomic DNA. The plasmid in the single colonies of the transformants in *E. coli* **(C)** and in *L. reuteri* **(D)**.

To validate the transcriptome results and demonstrate the protective role of these up-regulated genes against acid, bile salts and vacuum freeze-drying stress, overexpress the genes *csbD*, *osmC*, and *gatD* in *L. reuteri* Z204. These gene segments were cloned at the immediate downstream of the p32 promoter for overexpression in an *E. coli*-*L. reuteri* shuttle vector pMG36e (Figure 3A). Primer *osmC*-F/R, *csbD*-F/R, and *gatD*-F/R enabled the successful amplification of DNA fragments corresponding to three genes: *osmC*, *csbD*, and *gatD*, respectively (Figure 3B). These genes were effectively integrated into the pMG36e expression vector, transformed into DH5α, and the positive clone plasmids pMG36e-gatD/osmC/csbD were obtained by verifying the transformants using the universal 36-F/R primer (Figure 3C). These recombinant plasmids were then electroporated into *L. reuteri* Z204 in order to overexpress. Universal 36-F/R primer was used to confirm the existence of positive recombinant over-expressing bacteria (Figure 3D).

**Figure 4:**
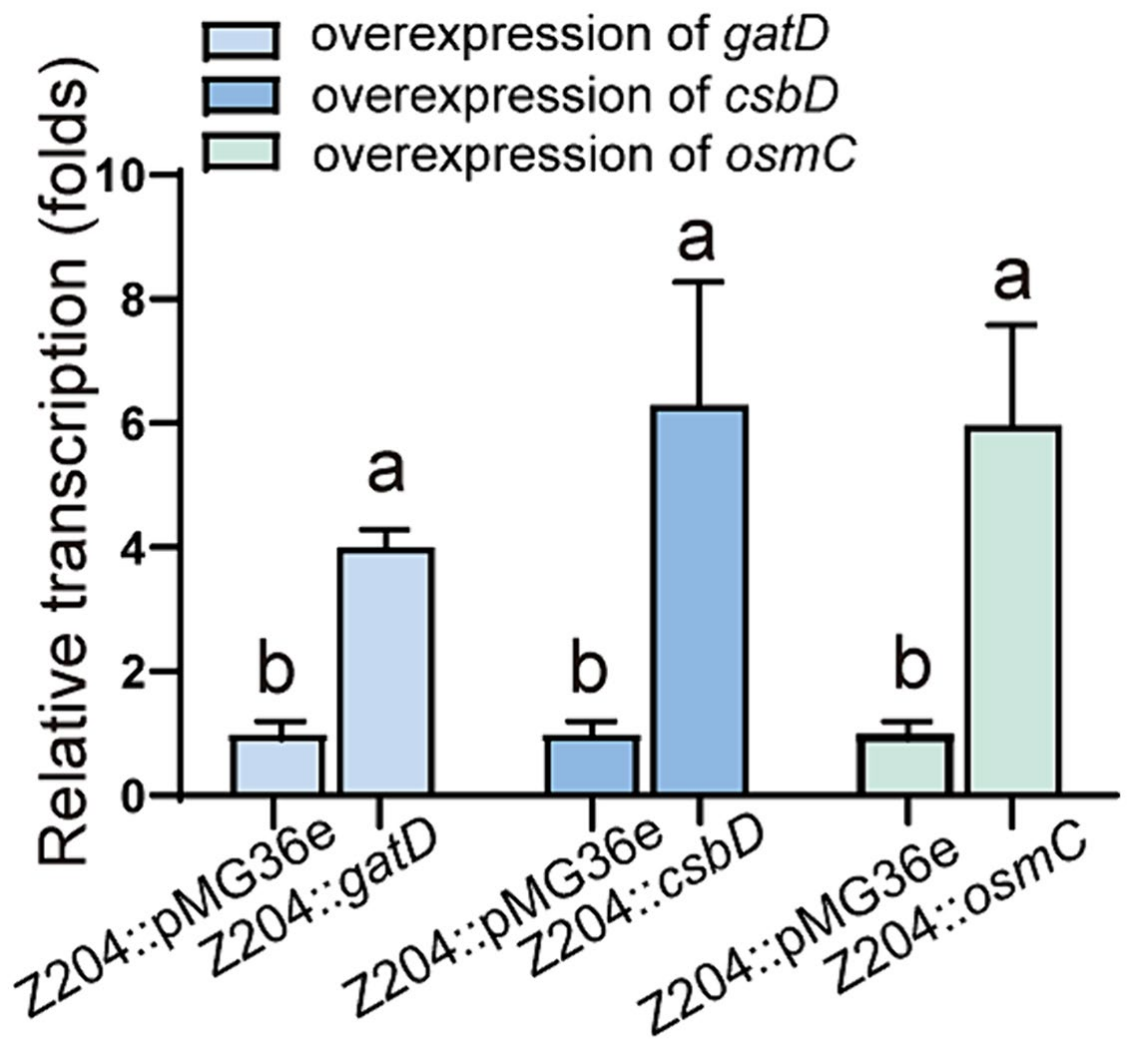
The mRNA levels of GatD, CsbD, and OsmC in the *L. reuteri* Z204 overexpressed strain measured by RT-qPCR.

To confirm the overexpression of genes encoding OsmC, CsbD, and GatD proteins, we chose to measure the transcription level of *osmC*, *csbD*, and *gatD* genes by RT-qPCR to determine whether they were overexpressed (*p* < 0.0001, *p* = 0.0096, *p* = 0.0059) (Figure 4), and further validated their expression at the protein level through TOF-MS analysis (Table 2). Based on the RT-qPCR and proteome results, all three genes were overexpressed in the overexpressed strain.

**Table 2:** Fold changes of overexpressed proteins in *L. reuteri* Z204 according to the proteome data.

Protein ID	Overexpressed strain vs. wt. (fold change)	Annotation
Integral component of membrane		
WP_003675030.1	5.23	CsbD family protein
Glutamine metabolism		
WP_003676161.1	9.98	Glutamine amidotransferase GatD (CobQ)
OsmC family protein		
WP_003669548.1	6.81	OsmC family protein

### Functional validation of stress resistance genes under acid, bile salts, and vacuum freeze-drying stress

**Figure 5:**
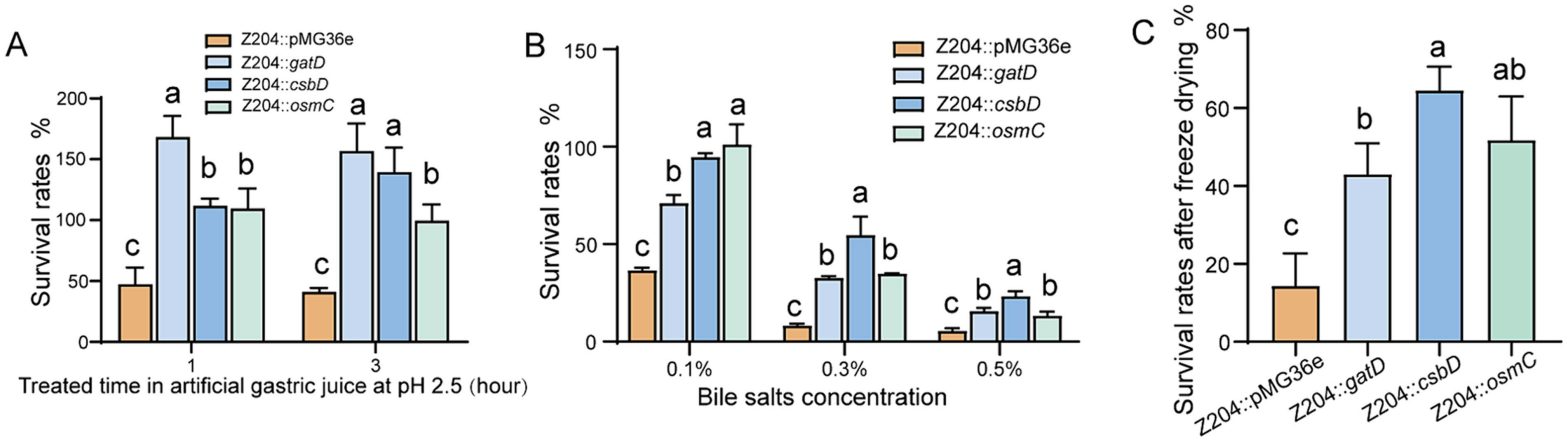
The *L. reuteri* Z204 overexpressed strains survival rates under the three stress conditions. The *L. reuteri* Z204 overexpressed strains survival rates under the artificial gastric juice stress **(A)**, bile salts stress **(B)**, and vacuum freeze-drying **(C)**. Error bars: standard derivations of three independent experiments. Data with different superscript letters (a, b, and c) are significantly different (*p* < 0.05) according to one-way ANOVA followed by Tukey’s test.

*Limosilactobacillus reuteri* strains overexpressing CsbD protein (Z204::*csbD*) were assessed for survival rates in artificial gastric juice, bile salts medium, and vacuum freeze-drying conditions. The overexpression of CsbD protein significantly enhanced *L. reuteri* Z204’s resistance to acid (Figure 5A), bile salts (Figure 5B), and its viability after freeze-drying (Figure 5C) in comparison to the control group Z204::36e strain. These findings imply that the membrane protein CsbD may act as a global regulator of *L. reuteri*’s cross-protection against acid, bile salts, and low-temperature treatment by modulating the membrane composition to provide cross-protection against low-temperature stress (Meneghel et al., 2017).

We experimented with artificial gastric juice, bile salts medium, and vacuum freeze-drying on *L. reuteri* strains overexpressing GatD. According to our research, overexpression of the GatD protein enhances *L. reuteri’*s resistance to acid tolerance (Figure 5A), increases its bile salts tolerance (Figure 5B), and improves its survival rate after freeze-drying (Figure 5C). These results imply that the glutamine amidotransferase GatD acts as a global regulator, providing *L. reuteri* with cross-protection against acid, bile salts, and low temperatures. Proton neutralization may be the mechanism by which GatD enhances resistance to salts and low-temperature drying stress; this mechanism is comparable to the glutamine-dependent acid tolerance mechanism that has previously been described (Lu et al., 2013).

It’s interesting to note that *L. reuteri* Z204 demonstrated enhanced acid resistance (Figure 5A), bile salts resistance (Figure 5B), and survival rate after freeze-drying (Figure 5C) upon overexpression of OsmC protein, which is involved in peroxide metabolism and oxidative stress defense (Si et al., 2019; Lyu et al., 2016). Our results may provide support to the hypothesis that OsmC, an osmotically generated bacterial protein, acts as a general regulator to provide *L. reuteri* with cross-protection against acid, bile salts, and low-temperature.

## Discussion

Immune homeostasis and physiology in the human gut are positively impacted by Lactic acid bacteria (Finamore et al., 2019; Meng et al., 2020). Furthermore, they protect the host by limiting the binding of pathogenic microbes and maintaining the balance between gut microbiota and lymphocytes (Riaz Rajoka et al., 2017). In order to influence host immunity and health, LAB are currently developed as fermented foods or probiotic powder (Daba and Elkhateeb, 2020; Wang et al., 2015; Wang et al., 2020). A key factor in establishing the characteristics of probiotics is their viability prior to technological processing and use (Bisutti and Stephan, 2020). Therefore, in order to prevent stress-related damage and increase the survival rate of linked products, it is crucial to comprehend the stress response and molecular control mechanism of LAB under various environmental conditions. Environmental harm may be lessened by LAB’s physiological and metabolic responses (Guan and Liu, 2020). Cross-protection, the ability to withstand both related and unrelated stresses, is a feature of adaptations seen under mild stress that can increase overall tolerance to different environmental pressures (Louesdon et al., 2014). Furthermore, it has the ability to stimulate cells within the global response signal, providing protection against external stressors (Papadimitriou et al., 2016). In order to resist multiple environmental stress conditions, also known as cross-protection (Gao et al., 2022), LAB exhibits numerous adaptive mechanisms by modulating their gene expression and signaling pathways, including up-regulation of response proteins, accumulation of suitable solutes, and regulation of membrane lipids (Zhen et al., 2020; Kulkarni et al., 2018). In the present study, overexpression of the membrane protein CsbD, the osmosis-induced bacterial protein OsmC, and the acid-related protein glutamate decarboxylase GatD, respectively, increased the acid tolerance (Figure 5A), bile salt tolerance (Figure 5B), and freeze-drying ability (Figure 5C) of *L. reuteri*. Through the expression of particular stress resistance proteins, the cross-protective characteristics of *L. reuteri* were shown to increase overall tolerance to numerous stimuli.

The cytoplasmic membrane is the main barrier for the majority of solutes and a major target of stress injury (Spano and Massa, 2006), research has demonstrated that sigmaB transcription factors triggered by metabolic stress control the membrane protein component csbD protein, which is a general stress response protein resistant to oxidative stress, protein denaturation, and osmotic stress (Prágai and Harwood, 2002; Akbar et al., 1999). In addition, the csbD protein was overexpressed in *Methyocystis* sp. strain SC2 (Han et al., 2017) and Group B *Streptococcus* (GBS) (Jia et al., 2023) in response to bile salt stress. Among these, in Group B *Streptococcus* (GBS), csbD promotes the transcription of several ABC transporter genes to excrete bile salts upon detecting bile salt stress. This enhances bacterial survivability in bile salts (Jia et al., 2023). As shown by our results, which showed that a large number of membrane proteins were up-regulated upon acid or bile salts stress (Table 1; Supplementary Table S1). *L. reuteri* Z204’s resistance to acid (Figure 5A) and bile salts (Figure 5B), as well as its survival rates after freeze-drying (Figure 5C), may be enhanced by overexpressing the membrane protein component CsbD protein. These results appear to indicate the hypothesis that the membrane protein CsbD functions as a global regulator of *L. reuteri*’s cross-protection against treatment with acid, bile salts, and low-temperature. This might be the consequence of either the regulation of global metabolic stress activators or the regulation of transport genes by csbD proteins. Altering the composition of the membrane, which can provide cross-protection against low-temperature stress may also have an impact on another cause (Meneghel et al., 2017). Many LAB improve stress resistance by altering the lipid composition of cytoplasmic membranes with various pretreatments (Papadimitriou et al., 2016). In order to prevent protons influx into cells from entering cells, LAB tends to increase the stiffness and density of the plasma membrane during acid stress, such as by modifying the content of fatty acids (van Der Heide and Poolman, 2000; Broadbent et al., 2010). By producing more unsaturated fatty acids, cyclic fatty acids, and branched-chain fatty acids, *L. acidophilus* has been shown to improve membrane fluidity under lactose starvation and display better tolerance to low-temperature stress (Wang et al., 2011).

The genes for glutamate decarboxylase (GAD), a pyridoxal phosphate (PLP)-dependent enzyme, are found in some *E. coli* and LAB strains (Barrett et al., 2012; Mazzoli and Pessione, 2016). By catalyzing the *α*-decarboxylation of L-glutamate or glutamate to produce gamma-aminobutyric acid (GABA), which helps bacteria in dealing with acid stress, GAD plays a crucial part in the glutamate-dependent acid resistance system (De Biase and Pennacchietti, 2012; Liu S. et al., 2022). Our findings showed that the glutamate decarboxylase GatD was up-regulated in response to acid stress (Table 1), which is consistent with a previous study’s finding that the GDA is crucial for the bacteria’s acid resistance system (De Biase and Pennacchietti, 2012). Otherwise, according to our findings, overexpression of the acid-associated protein glutamate decarboxylase GatD might increase *L. reuteri’*s bile salts tolerance (Figure 5B), survival rate after freeze-drying (Figure 5C), as well as its ability to withstand acid (Figure 5A). It’s interesting to note that the strain treated with artificial gastric fluid grew better than the untreated strain in the acid tolerance test of the overexpressed GatD strain used in this work. This phenomenon could be caused by: despite the fact that the properties of GADs differ significantly between LAB species and strains, most LAB GADs are significantly more active in acidic environments than in neutral pH (Cui et al., 2020). Consequently, the GatD overexpression strain’s glutamate decarboxylase GatD was able to deacid L-glutamate or glutamate into GABA, which gave the strain more energy for growth than it would have without treatment (Mazzoli and Pessione, 2016). As a consequence, the acid-treated strain grew more better than the untreated strain. According to these results, *L. reuteri*’s tolerance to acid, bile salts, and low-temperature is controlled globally by the glutamate decarboxylase GatD, which acts as a global regulator to cross-protection the acid, bile and low-temperature tolerance. By altering the proton content in the cells, the GatD may be able to improve resistance to salts and low-temperature drying stress.

Oxidative stress is brought on by the large-scale generation of reactive oxygen species (ROS) by LAB during fermentation. ROS (superoxide and hydroxyl radicals) can have a deleterious effect on microbial components as proteins, lipids, DNA, and RNA during oxidative stress (Larasati et al., 2018). *Thermus thermophilus* (Rehse et al., 2004), *Thermococcus kodakarensis* KOD1 (Park et al., 2008), *E. coli* (Shin et al., 2004; Lesniak et al., 2003), *Mycobacterium tuberculosis* and *M. smegmatis* (Saikolappan et al., 2011), have all been found to contain the OsmC protein, which has hydroperoxide peroxidase properties and can shield microorganisms from oxidative damage by lowering peroxide substrates. Furthermore, *Thermococcus kodakarensis* KOD1 overexpressed OsmC in order to fend off oxidative damage brought on by high osmotic pressure when exposed to salt (Park et al., 2008). Interestingly, both acid and bile salt stressors were shown to increase the expression of the osmotically induced bacterial protein OsmC in this study (Table 1). Additionally, *L. reuteri* Z204’s resistance to acid (Figure 5A), bile salts (Figure 5B), and freeze-drying (Figure 5C) could all be enhanced by overexpression of the OsmC protein. These results seem to verify the idea that OsmC, an osmotically induced bacterial protein, functions as a general regulator of *L. reuteri* cross-protection against oxidative stress exposure to acid, bile salts, and low-temperature.

Understanding the adaptive response mechanism under adverse conditions is crucial for optimizing the performance of LAB during production and downstream application (Derunets et al., 2024). Theoretically, new strategies to increase the survival rate of LAB under different stresses can be developed with a thorough grasp of the molecular mechanisms of environmental stress adaptation in lactic acid bacteria and appropriate protective measures based on the stress response mechanisms. In addition, a methodical comprehension of the “stressome” of LAB and the development of strains that enhance tolerance via cross-protection—particularly alterations in critical enzyme activities—will offer valuable new approaches for the focused screening or molecular modification of stress-resistant LABS in the fields of environmental and industrial protection (Gao et al., 2022). In this case, creating an acid-tolerant module made up of the transcription factor of the AR system (*gadE*), a periplasmic protein chaperone (*hdeB*), and two reactive oxygen scavenging enzymes (*sodB* and *katE*) may increase the growth robustness and productivity of industrial *E. coli* in mildly acidic environments (Yao et al., 2022). Enhancing the stress resistance of LAB through pre-adaptation (Bisson et al., 2023; Bommasamudram et al., 2023), genetic modification of strains (Yao et al., 2022), and modification of culture conditions (Hao et al., 2021) are particularly essential for decreasing production costs, stabilizing product quality, and enhancing the survival and vitality of strains used in industrial production. This will help us make the most use of lactic acid bacteria as starter cultures and probiotics in industrial production.

Genetic manipulation based on the factors and signaling pathways regulating bacterial stress response has drawn special interest among molecular mechanisms of how LAB responds to stressful situations. In this study, the efficacy of *L. reuteri’s* resistance to acid resistance, bile salts resistance, or freeze-drying was improved by overexpressing *csbD*, *osmC*, and *gatD*. Overall, GatD was most successful in enhancing acid tolerance, whereas CsbD was most effective in improving bile salts tolerance. Furthermore, it was found that CsbD was the most successful in increasing freeze-drying tolerance, followed by OsmC. These findings enhance our understanding of the molecular mechanism adopted by *L. reuteri* in response to acid, bile salts, and freeze-drying stress. In order to promote global cross-protection, these findings suggest a novel practical strategy to improve resistance to acid, bile salts resistance, or freeze-drying stress function. They also show that LAB respond to environmental stress and that cross-protection strategies can increase resistance to extremely high levels of environmental stress. This establishes the framework for further investigations on the LAB stress mechanisms that provide cross-protection.

## Data Availability

The datasets presented in this study can be found in online repositories. The names of the repository/repositories and accession number(s) can be found in the article/Supplementary material.
